# Digital Biomarkers of Cytokine Release Syndrome: Scoping Review and Ontology Development of the Role and Relevance of Digital Measures Using a Mixed Methods Approach

**DOI:** 10.2196/71956

**Published:** 2025-12-11

**Authors:** Christopher J Medberry, Charlotte Zoe Angel, Ashita S Batavia, Sarah Bradley, Nunzio Camerlingo, Melinda Chen, Mohamed Datoo, Premal Kamdar, Erik Koenig, Lieke Kusters, Celine Marquez, Ujwani Nukala, Michael Pettinati, Ruth Phillips, Daniel P Sanchez, Nazila Shafagati, Jenifer Siegelman, Mark Stewart, Cindy Varga, Benjamin Vandendriessche, Matt Wilkes, Dana L Wolff-Hughes, David Zahavi, Sylvain Zorman, Samantha J McClenahan

**Affiliations:** 1 Johnson and Johnson Innovative Medicine, New Brunswick, NJ, United States; 2 Fosanis GmbH, Berlin, Germany; 3 Blue Spark Technologies, Inc, Westlake, OH, United States; 4 Pfizer, Inc, Cambridge, MA, United States; 5 Takeda (United States), Cambridge, MA, United States; 6 Bristol Myers Squibb, Princeton, NJ, United States; 7 Roche (Switzerland), Basel, Switzerland; 8 Genentech, San Francisco, CA, United States; 9 Center for Biologics Evaluation and Research, US Food and Drug Administration, Silver Spring, MD, United States; 10 ActiGraph, Pensacola, FL, United States; 11 The Sidney Kimmel Comprehensive Cancer Center, Johns Hopkins Medicine, Baltimore, MD, United States; 12 Friends of Cancer Research, Washington, DC, United States; 13 Department of Hematologic Oncology and Blood Disorders, Levine Cancer Institute, Charlotte, NC, United States; 14 Digital Medicine Society, 90 Canal Street, 4th Floor, Boston, MA, 02114, United States, 1 765-234-3463; 15 Department of Electrical, Computer, and Systems Engineering, Case Western Reserve University, Cleveland, OH, United States; 16 Current Health Inc, Dover, DE, United States; 17 National Cancer Institute, Bethesda, MD, United States

**Keywords:** cytokine release syndrome, immunotherapy, digital health, scoping review, digital biomarkers, PRISMA, Preferred Reporting Items for Systematic Reviews and Meta-Analyses

## Abstract

**Background:**

Advancements in cancer-targeted immunotherapies have transformed care, yet these therapies present a high likelihood of cytokine release syndrome (CRS), a potentially severe immune-related adverse event. The ability to identify CRS earlier could improve care by mitigating risks, widening patient access, and reducing the burden on patients, caregivers, and health care providers. Digital health technologies (DHTs) are promising for early CRS detection by enabling continuous measurement of vital signs before symptoms are detected through standard intermittent clinical assessments. While the number of studies is increasing, inconsistencies in the symptoms and measures strongly associated with CRS highlight the need for a comprehensive review to identify the most reliable and commonly reported indicators. Despite this growing body of research, reliable predictive and diagnostic measures for early warning for CRS following the administration of immunotherapy have yet to be established.

**Objective:**

This scoping review aims to address this gap by developing an ontology of early warning signs for CRS—a structured model defining measurement concepts, properties, and interrelationships—for advancing early warning models for CRS.

**Methods:**

We conducted a mixed methods study including a scoping literature review, surveys, and interviews. The literature review searched PubMed and Embase (last searched March 19, 2024) for articles reporting measures collected between therapy administration and CRS onset and linked to CRS onset. Studies were limited to publications between January 2014 and March 2024, excluding those that did not assess an immunotherapy-based treatment, were not conducted in humans, did not compare collected measures to CRS diagnosed using standard of care, or were not available in English. Identified measures were further assessed through surveys and interviews with subject matter experts (SMEs; n=22) and key opinion leaders (KOLs; n=8) and analyzed using qualitative and quantitative methods.

**Results:**

Thirty studies met eligibility criteria and used a variety of grading scales and thresholds for severe CRS. A comprehensive ontology of early warning signs for CRS that includes physiological signs, clinical symptoms, and laboratory markers was developed. Within the full ontology, a common set of early warning signs for CRS—temperature, heart rate, blood pressure, and oxygen saturation—was identified as the minimally necessary data to evaluate for their predictive value for CRS. Three of these 4 signs align with the American Society for Transplantation and Cellular Therapy (ASTCT) criteria for CRS grading and other clinical grading scales for CRS.

**Conclusions:**

Standardization and adoption of the ontology of early warning signs for CRS will streamline data collection to support the creation of robust, fit-for-purpose datasets. This approach ensures practical and informative data collection, ultimately enhancing the ability to predict and manage CRS effectively. Developing predictive models based on these early warning signs can enhance CRS risk assessment, support decentralized trials, and improve access to cancer-targeted immunotherapies.

## Introduction

Cancer-targeted immunotherapies, such as Chimeric Antigen Receptor (CAR) T-cell therapies, bispecific antibodies, and checkpoint inhibitors, have made a profound impact on the treatment of oncology patients but may cause adverse events. Cytokine release syndrome (CRS) is a complex immune response characterized by the rapid release of cytokines into the blood by immune cells affected by the immunotherapy [1]. CRS incidence, onset, and severity vary by therapy, with CAR T-cell therapies having high rates of severe CRS compared to bispecific antibodies [2-4]. Since severe CRS (grade 3 or higher) can result in multiorgan failure and death, early detection and timely intervention are essential and have the potential to reduce clinical trial and health care costs associated with hospitalization and intensive care management, together promoting the broader adoption and accessibility of these innovative therapies [5,6]. Importantly, improved early detection has the potential to enhance patient outcomes by enabling prompt treatment that can mitigate severe symptoms, reduce complications, and improve survival. Furthermore, the availability of effective methods for early CRS detection and intervention may expand the use of these highly effective therapies into diverse care settings, such as community oncology clinics [7,8].

Several CRS grading scales have been developed to standardize the assessment of CRS severity, including the American Society for Transplantation and Cellular Therapy (ASTCT) consensus grading, the Penn scale, the Lee scale, the CAR T-Cell Therapy–Associated TOXicity (CARTOX) system, and the Common Terminology Criteria for Adverse Events (CTCAE) [9-13]. The existing CRS grading scales use intermittently measured physiological signs, laboratory markers, and clinical symptoms to assess CRS severity for clinical decision-making [10-12,14]. For example, the ASTCT scale focuses on 3 core physiological signs (fever, hypotension, and hypoxia), aiming to streamline grading across institutions. The Penn and Lee scales incorporate broader clinical features and, in some cases, require more detailed assessments, such as organ dysfunction. Similarly, the CARTOX system includes neurologic symptoms in addition to CRS grading, with an emphasis on CAR T-cell therapy–associated toxicity. CTCAE, while widely used in oncology, has been adapted for CRS but may lack specificity for immunotherapy-related presentations.

Importantly, these grading scales are not used for early detection but rather are used postdiagnosis to assess CRS severity. These scales may miss early changes in the development of the syndrome and do not provide predictive insights into whether a patient will progress to severe CRS, restricting their clinical usage in guiding timely interventions.

Efforts to identify warning signs using digital health technologies (DHTs) have shown success in early CRS detection [15]. In a recent study, wearable sensors detected CRS episodes with a median lead time of 7 hours (IQR ~5.4-8.6) before recognition by standard nursing care, with patients maintaining a median adherence rate of 71% (IQR 55‐84) during the high-risk period [16]. Similarly, in patients receiving bispecific antibodies, remote monitoring detected significant pre-CRS changes in temperature (+1.7°C), heart rate (+16.66 beats per minute [bpm]), and respiratory rate (+1.86 respirations per minute [rpm]) up to 2 hours before detection by standard care and identified fever events nearly 5 hours earlier on average, including several missed entirely by conventional monitoring [17].

DHTs are a broad range of emerging technologies using hardware, software, and computing platforms that offer a promising opportunity to enable early CRS warning, in part, through the real-time and remote capture of continuous physiological parameters [18]. Their adoption in clinical settings has grown steadily, with physician use of remote patient monitoring devices increasing from 12% in 2016 to 30% in 2022, reflecting a broader shift toward technology-enabled care delivery [19]. These data streams can be significantly more granular than traditional data measurements and support advanced analytics for detection of early warning signs by capturing continuous, high-frequency data that offers a more detailed and dynamic view of a patient’s condition.

DHTs can produce rich datasets; however, there is no standardization about what and how their data are reported for CRS. Additionally, because higher grades of CRS are less common events, data aggregation will be essential to create a sufficiently large, fit-for-purpose dataset that can overcome the challenge of data imbalance and enable development of early warning models. To achieve this, there is a need for standardization and uniform data collection during the immediate postimmunotherapy window. This research uses a scoping review and expert engagement to collate and evaluate potential early warning signs for CRS in oncology patients. Results were assembled into an associated ontology for use by researchers developing early warning models that are compatible with inpatient, outpatient, and hospital-at-home settings.

## Methods

### Overview

A mixed methods approach was used to construct the ontology of measures of early warning for CRS, combining quantitative data from a scoping review of peer-reviewed studies with qualitative expert insights derived from surveys, interviews, and focus groups to ensure a comprehensive and evidence-based framework for identifying early warning signs [20]. A mixed methods design was chosen to enhance the breadth and depth of the findings, with the peer-reviewed studies identifying reported CRS measurements, which were assessed for their relationship to early CRS through engagement with CRS experts.

### Scoping Review

PRISMA (Preferred Reporting Items for Systematic Reviews and Meta-Analyses) guidelines for scoping reviews were followed (Checklist 1) [21]. As a scoping review, this work did not meet the criteria for registration on PROSPERO (International Prospective Register of Systematic Reviews) [22]. The protocol is available from the corresponding author.

PubMed and Embase databases were searched for peer-reviewed articles between January 2014 and March 2024. The literature search used search terms as follows (1) medical subject heading (MeSH) term for human participants; (2) keywords for CRS including cytokine storm; (3) keywords for immunotherapy; (4) keywords for measures, including signs, biomarkers, and symptoms; and (5) keywords related to timing of measures including onset, early, and predict. The complete search string is provided in Multimedia Appendix 1.

All articles identified in our search strategy underwent a two-stage screening process: (1) title and abstract screening and (2) full text screening based on the population, intervention, control, and outcome (PICO) criteria outlined in Table 1. Studies that were not available in English were excluded during the title and abstract screening process. To ensure the approach’s sensitivity and specificity in identifying relevant articles, 2 independent investigators began by screening a random selection of 20% of publications; disagreements were resolved by consensus, and clarifications were made using the eligibility criteria in relation to the order of exclusion metrics and timing of measure collection. It was determined a priori that agreement of ≥90% among the sample would support a single investigator extraction for the remaining publications. To ensure continued reliability, a second investigator conducted periodic quality checks on approximately 10% of the remaining publications during the screening and extraction process, maintaining interrater agreement above 90%. Disagreements were resolved through discussions between reviewers.

**Table 1.** Study selection eligibility criteria. Description of the patient, intervention, control, outcome (PICO) criteria [23].

Criteria	Inclusion	Exclusion
Patient	Patients undergoing immunotherapy treatment and monitored for Cytokine Release Syndrome (CRS)	Studies that do not report data collected from human participants; studies that do not report cases of CRS
Intervention	Immunotherapy	Studies that do not assess an immunotherapy
Comparator	Any	None
Outcome	Early warning signals for the onset of CRS	Studies that do not report data on early signals of the onset of CRS defined as the time postimmunotherapy up to the diagnosis of CRS; studies that do not relate measures collected to CRS (ie, measures for overall response rate of immunotherapy)

A systematic data extraction process was used to construct the ontology. Extracted fields included publication record, CRS reporting, immunotherapy, reported measures and procedures, and study design and sample characteristics. Data extraction was either predefined categorical coding or open text, in which full text could be input to fulfill the response and minimize error. All data were descriptively analyzed, with frequency counts and percentages used to map study characteristics and reported measures. Bar graphs were generated to visualize key patterns and trends.

### Focus Group

A group of CRS subject matter experts (SMEs; n=22), including clinicians specializing in oncology and immunotherapy, regulatory scientists, digital health researchers, and industry leaders with expertise in CRS and immunotherapy, and safety were convened from the Digital Health Measurement Collaborative Community (DATAcc), a collaborative community hosted by DiMe with the Food and Drug Administration’s Center for Devices and Radiological Health, to assess the measures of early warning for CRS identified through the scoping review [24,25]. A survey was deployed to (1) corroborate CRS measures identified in the scoping review and (2) evaluate early warning signs for outpatient monitoring (Multimedia Appendix 1). Specifically, a Likert scale was used to measure the level of agreement or disagreement in the clinical measures identified from the scoping review for their clinical usage in assessing CRS, digital readiness, and predictive value. Qualitative questions were incorporated to allow for the identification of missing clinical measures not captured in the scoping review and to provide evidence for selection choices. The survey results, including deidentified responses for measures of inclusion and exclusion, were discussed in 3 focus group sessions (one main and 2 follow-up sessions) and used to collaboratively develop criteria for including measures in the ontology. Measures that were not selected by any SME were not considered for the ontology. Any measure selected by at least one SME or any new measure identified by >10% of SMEs was assessed for inclusion in the ontology. Consensus was reached if a measure was selected by >70% of SMEs with less than 15% disagreement, as used in other consensus processes [26]. The ontology was initially constructed by incorporating the survey data with findings of the scoping review, followed by iterative one-week rounds of refinement of the classes, concepts, properties, and values with focus group participants.

### Interviews and Qualitative Analysis

Semistructured interviews were conducted with 8 key opinion leaders (KOLs) who have direct experience managing CRS (5 primary practice oncologists, 2 patient advocates, and a clinical trialist) to assess the identified measures from the scoping review and draft ontology from the focus group. KOLs were purposively sampled for their expertise in CAR T-cell and bispecific programs and were distinct from the SMEs engaged in the focus group. Interviews were conducted over Zoom (Zoom Video Communications, Inc) by a single interviewer in April and May 2024. The data collected during the interviews included direct feedback on the relevance and applicability of the proposed measures for the selected time frame, as well as suggestions for additional features or modifications, such as definitions, measurement values, and interrelationships between concepts (Multimedia Appendix 1).

Interview responses were transcribed by 2 reviewers and analyzed using Braun and Clarke’s 6-phased framework for thematic analysis [27]. A deductive thematic approach was used with initial themes derived a priori from the identified early warning signs and their associated properties, values, and definitions. The analytic process involved repeated readings of transcripts for familiarization, coding of transcripts into ontology and measure categories and features, and collation of codes into candidate themes. Themes were iteratively reviewed against coded extracts and entire data sets, refined to capture their analytic scope, and finalized through team discussion.

Manual coding was conducted using basic word processing tools with discrepancies resolved through discussions. Interviews were conducted and coded by researchers from DiMe with expertise in digital health technologies and evidence synthesis, but without clinical expertise in CRS. This outsider perspective reduced the likelihood of imposing clinical assumptions during analysis, while reflexivity was maintained by documenting analytic decisions and triangulating findings with clinical experts. Rigor was supported through confirmability, dependability with documented procedures, credibility through expert validation, and transferability through detailed descriptions, in line with Braun and Clarke [28]. The findings from all interviews were synthesized, and the themes were reported to the focus group of SMEs to inform revisions of the ontology. Final themes were validated by presenting to the focus group for debrief and review. This deductive approach allowed us to evaluate the clinical relevance, feasibility, and patient-centeredness of proposed CRS early warning signs and to refine the draft ontology.

### Ethical Considerations

This study involved surveys and interviews with SMEs and KOLs to collect expert insights relevant to the research aims. No personal or sensitive information was collected or analyzed. In accordance with institutional policies and federal regulations, the project was determined to be exempt from human participants regulations and formal institutional review board approval was not required [29]. Informed consent was obtained from all participants prior to the survey and interviews. Participants were informed about the study purpose and their voluntary participation, with the option to withdraw at any time. No compensation was provided for participation. All data were anonymized and confidentiality was maintained throughout the study. No identifiable participant information is included in the manuscript or supplementary materials.

## Results

### Early Warning Signs in the Literature

**Figure 1.**
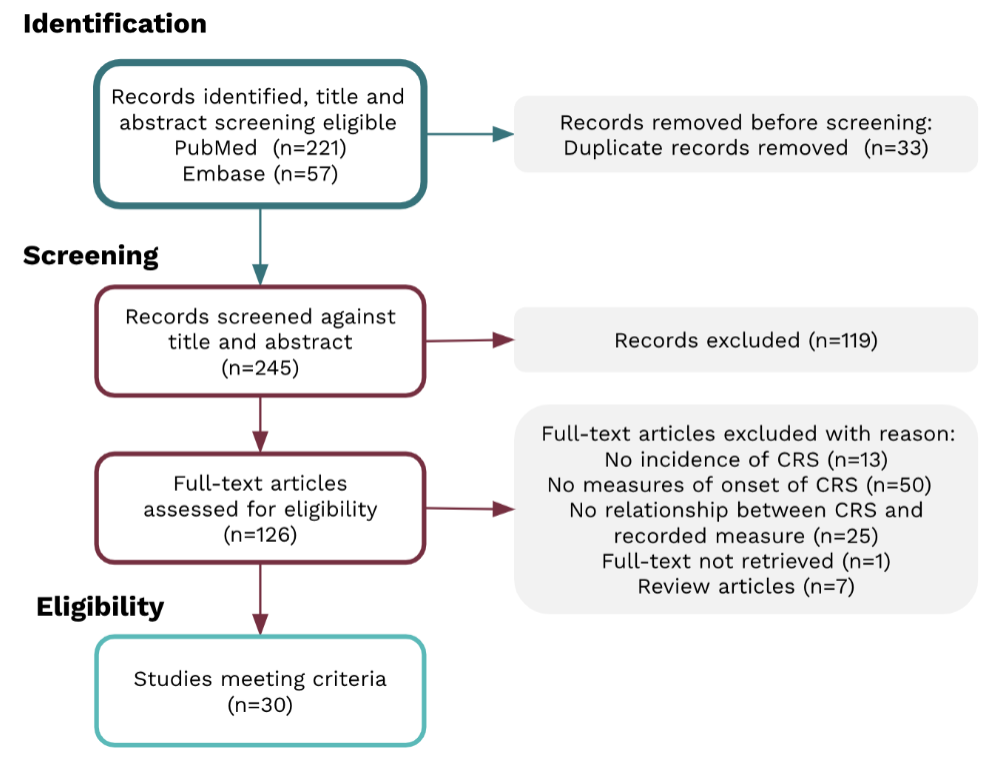
Preferred Reporting Items for Systematic Reviews and Meta-Analysis flow diagram of the scoping review process. PRISMA: Preferred Reporting Items for Systematic Reviews and Meta-Analysis; CRS: cytokine release syndrome.

278 studies were identified during the literature search; 33/278 (11.9%) duplicates were removed. In total, 126/278 (45.3%) full-text studies were obtained following title and abstract screening. Around 30/126 studies (23.8%) met the eligibility criteria for review. The main reasons for exclusion were no reported cases of CRS, measures collected outside the timeframe of interest or not collected in relation to CRS, or the publication being a literature review (Figure 1).

**Figure 2.**
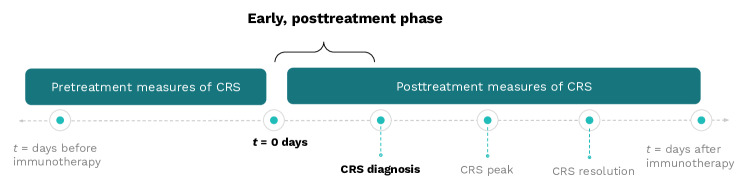
Timing of measure selection. To identify measures of CRS with early warning value, the literature review captured measures collected from the administration of immunotherapy to the onset of CRS, as defined by a clinical diagnosis of Grade 1 or higher. CRS: cytokine release syndrome.

**Figure 3.**
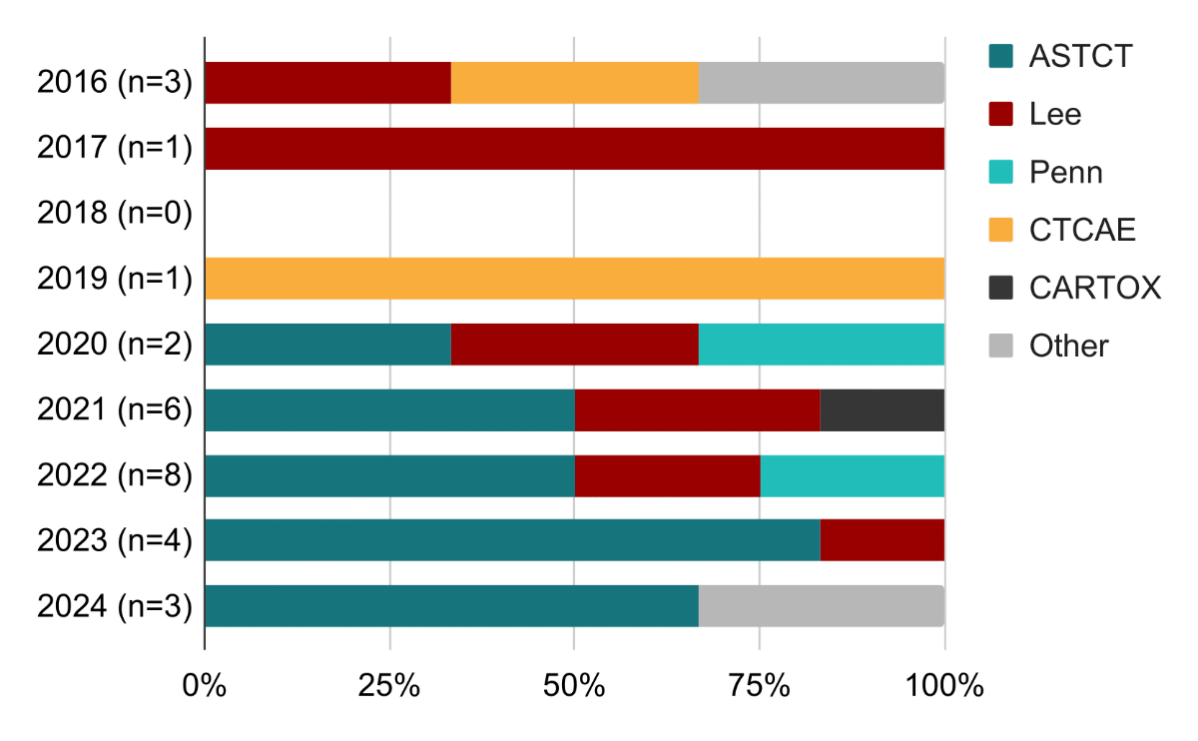
Report of cytokine release syndrome by grading scale. Distribution of the grading scales used based on publication date of the studies, supporting an uptake of ASTCT grading scale following its release in 2019. ASTCT: American Society for Transplantation and Cellular Therapy; CARTOX: CAR T-Cell Therapy–Associated Toxicity; CTCAE: Common Terminology Criteria for Adverse Events.

The 30 eligible studies were published between 2016 and 2024 (Table S1 in Multimedia Appendix 2) [30-59]. The studies reported measures collected between the administration of immunotherapy and the onset of CRS, which was defined by multiple grading scales (Figure 2). Clinically, the use of different grading scales can lead to variability in severity classification, affecting treatments and comparable outcomes across clinical research and clinical care [12]. A majority of studies used the American Society for Transplantation and Cellular Therapy (ASTCT) grading scale, with an increase in adoption following its release in 2019 (Figure 3). Eighteen studies [30-32,34,37,38,41,42,44,46-48,50,52,53,56,57,59] reported CRS by severity with severe CRS indicating grade 3 or greater, while 9 studies [33,35,39,40,43,45,49,54,58] referred to cases as severe CRS but did not indicate a CRS grade.

Measures were broadly classified into 3 categories: laboratory markers, physiological signs, and clinical symptoms (Table 2). Cytokines [30-33,36,37,40,41,44,46,49-54,56,58,59] and temperature [30,32,37-39,41-45,47-52,54-56,58] were the most common measures reported in 20 of the 30 articles. Cytokines were derived from either serum or plasma, typically as part of a panel, and were grouped into a single category for this study. Measurement of temperature and the report of a fever were grouped together as a physiological measurement based on the assumption that a report of a fever required a physical temperature recording. Clinical symptoms were reported in 20% of the articles.

**Table 2.** Study-reported measures captured between the administration of immunotherapy and the onset of cytokine release syndrome [30-59].

Measures by category	Studies, n
Physiological signs	
Temperature or fever	20
BP^a^	7
SpO_2_^b^	7
HR^c^	6
RR^d^	2
Clinical symptoms	
Chills	4
Fatigue	1
Headache	3
Myalgia	1
Vomiting	2
Malaise	1
Confusion	1
Dyspnea	1
Rash	1
Laboratory markers	
Cytokines	20
CRP^e^	19
Ferritin	11
CAR-T^f^ transgene copy numbers	2
White blood cell count	2
Presepsin	1
Procalcitonin	1
Neutrophils	1
Lymphocytes	1
ALT^g^	3
AST^h^	2
ALP^i^	1
Bilirubin	3
Albumin	2
Prothrombin time	2
Creatinine	5
Blood urea nitrogen	1
Hemoglobin	2
Platelets	7
Fibrinogen	2
Fibrin	1
LDH^j^	6

a BP: blood pressure.

b SpO_2_: saturation of peripheral oxygen.

c HR: heart rate.

d RR: respiratory rate.

e CRP: C-reactive protein.

f CAR-T: Chimeric Antigen Receptor T.

g ALT: alanine transaminase.

h AST: aspartate aminotransferase.

i ALP: alkaline phosphatase.

j LDH: lactate dehydrogenase.

### Selection of Measures With Early Warning Value in CRS

The selection and refinement of early warning signs for CRS were achieved through a comprehensive mixed methods approach, integrating findings from a scoping literature review with qualitative and quantitative insights from SMEs (n=22) and KOLs (n=8). This iterative process focused on identifying the most reliable indicators to support the development of an ontology for early CRS detection. The integration of survey findings, focus group discussions, and thematic analysis of KOL interviews yielded four overarching themes that guided refinement of the CRS ontology.

#### Theme 1: Identification and Categorization of Measures That Inform Early CRS Detection

The survey identified 18 measures from the scoping review deemed likely to support early warning for CRS (Tables3  4). Additionally, 2 new measures—activity level and heart rate variability—were recognized by over 10% of SMEs as measures with potential to provide early warning value for CRS. These measures were not identified in the peer-reviewed literature covered in our scoping review, highlighting a current evidence gap, though the measures have been noted in gray literature and early-stage digital health research [17]. Key points from the focus group discussion included (1) no measures should be excluded unless there was clear and substantial disagreement, and (2) those measures not sourced from peer-reviewed articles should be clearly notated. Accordingly, measures were categorized as primary (consensus-based), secondary (less than 15% disagreement), or emerging (identified by >10% of SMEs but lacking peer-reviewed evidence).

**Table 3.** Outcome to measure and properties of the common set of early warning signs for cytokine release syndrome.

Outcome to measure and property	Value	Use of property	Continuously collected data: binned
Fever generation			
Rise in body temperature	°C over time or °F over time	Optional	Median, max^a^, min^b^, IQR
Core body temperature			
Baseline	°C or °F	Recommended	Median, max, min, IQR
Current	°C or °F	Required	Median, max, min, IQR
Threshold	°C or °F	Recommended	max, min
Hypoxia			
	(SpO_2_)^c^ % over time	Optional	Median, max, min, IQR
Oxygen saturation			
Baseline	(SpO_2_) %	Recommended	Median, max, min, IQR
Current	(SpO_2_) %	Required	Median, max, min, IQR
Threshold	(SpO_2_) %	Recommended	max, min
Respiration rate			
Baseline	rpm^d^	Optional	Median, max, min, IQR
Current	rpm	Optional	Median, max, min, IQR
Threshold	rpm	Optional	max, min
Hypotension			
Drop in blood pressure	mm Hg over time	Optional	Median, max, min, IQR
Blood pressure (systolic and diastolic)			
Baseline	mm Hg	Recommended	Median, max, min, IQR
Current	mm Hg	Required	Median, max, min, IQR
Threshold	mm Hg	Recommended	max, min
Tachycardia			
Rise in heart rate	bpm^e^ over time	Optional	Median, max, min, IQR
Heart rate			
Baseline	bpm	Recommended	Median, max, min, IQR
Current	bpm	Required	Median, max, min, IQR
Threshold	bpm	Recommended	max, min
Measurement interval			
Interval between measurements^f^	sec^g^, min^h^, h^i^	Required	bin at 1h intervals
Time postimmunotherapy	sec, min, h	Required	—^j^
Start and end	Timestamp	Required	—
Minimum number of recordings	Number	Required	—
Metadata			
Individual-specific variables (eg, age)	Varies (variable-dependent)	Recommended	—
Environmental variables (eg, location of use)	Varies (variable-dependent)	Recommended	—
Context-of-use dependent (eg, disease)	Varies (variable-dependent)	Required	—
Measurement modality (eg, form factor)	Varies (variable-dependent)	Required	—

a max: maximum.

b min: minimum.

c SpO_2_: peripheral capillary oxygen saturation.

d rpm: respirations per minute.

e bpm: beats per minute.

f Interval between measurements for on-demand collections should not exceed 4 hours during awake hours.

g sec: second.

h min: minute.

i h: hour.

j Not applicable.

**Table 4.** Measures to aid the common set of early warning signs of cytokine release syndrome. This table outlines supplementary measures that enhance the detection and monitoring of cytokine release syndrome beyond the core early warning signs.

Classes and measures		Values
Inflammatory markers	
C-reactive protein (CRP)		Mass per volume (eg, pg/mL^a^, mg/dL)^b^
Ferritin		Mass per volume (eg, pg/mL, mg/dL)
Cytokines	
Interleukins (eg, IL-6)^c^		Mass per volume (eg, pg/mL, mg/dL)
Endothelial damage	
Lactate dehydrogenase (LDH)		U/L
Platelet count		counts/L
Constitutional symptoms	
Chills		Verified instrument
Fatigue		Verified instrument
Headache		Verified instrument
Myalgia		Verified instrument
Dyspnea		Verified instrument
Vomiting		Verified instrument
Emerging measures	
Activity level		Time spent inactive, step count, etc
Heart rate variability (HRV)		Validated HRV measure
Skin temperature		°C or °F

a pg/mL: picograms per milliliter

b mg/dL: milligram per deciliter.

c IL-6: interleukin-6.

Across focus groups and interviews, there was broad agreement on a core set of early warning signs and clinical characteristics that are essential for early warning of CRS. Temperature or fever emerged as the most sensitive early indicator.

For CAR T[-cell] patients, even modest temperature elevations (e.g., 99°F) can trigger re-evaluation due to neutropenia risk, whereas for bispecific therapies, fever may be combined with other factors for clinical decision-making.

[Interviewee 1]

Blood oxygen saturation and blood pressure were identified as key measures for higher-grade CRS, while heart rate was indicated as an important early indicator, even though it is not consistently included in current grading scales.

Unanimous agreement was reached for the inclusion of the identified laboratory markers and physiological signs; however, perspectives diverged on the relationship between select clinical symptoms and CRS. The primary concern was discerning the difference between CRS and other adverse events, mainly immune effector cell-associated neurotoxicity syndrome (ICANS), a neurological syndrome that sometimes coincides with CRS or follows CRS.

The delineation of ICANS and CRS is difficult to define—they’re not perfectly separated.

[Interviewee 6]

I would remove headache and confusion. Those are ICANS symptoms, and I would treat them differently.

[Interviewee 1]

Recommendations were made for the removal of confusion and headaches by 5 oncologists and a clinical trialist and 4 out of 5 oncologists, respectively, as these symptoms were deemed to be clinical symptoms that were more informative of ICANS. Two interviewed oncologists also recommended removal of the rash, indicating it was not a classical symptom or a symptom associated with CRS.

After 2 SME follow-up sessions, consensus was reached to remove confusion and rash from the CRS early warning signs with no recommendations for inclusion of either measure by an SME following the second discussion. Consensus was not reached, however, for the removal of headache, and it was retained as a secondary measure.

Temperature was further defined into 2 distinct components to distinguish between different physiological processes (1) core body temperature, which is tightly regulated; the normal range (36.1°C to 37.2°C) is typically maintained regardless of environmental temperature changes, and (2) skin temperature, which can fluctuate heavily in response to surrounding changes in temperature and the patient’s activity level [60]. The former is an essential component of CRS, while the relationship of the latter is less clear.

#### Theme 2: Timing and Relative Changes Provide Critical Insight Beyond Fixed Thresholds

Findings from both focus groups and interviews emphasized that the clinical usage of early warning signs depends on how and when they are measured. Personalized baselines were consistently preferred over absolute thresholds, as relative changes were considered more informative than static cutoffs.

Heart rate is really more patient-specific. If I’m normally at 55, jumping to 85 is huge. If a patient is normally at 85 and hits 95, it may not be as big of an issue.

[Interviewee 3]

Blood oxygen saturation and blood pressure were recognized for higher-grade or later-onset CRS, with suggested thresholds (SpO_2_ <90%, systolic BP <90‐100 mm Hg) reflecting clinical concern for rapid deterioration. Continuous or high-frequency monitoring was favored over intermittent assessments, particularly for temperature, as it allowed for more precise identification of clinical changes and treatment initiation points.

Monitoring should occur every 4 to 6 hours. Anything less than 4 times a day makes me worried.

[Interviewee 5]

There should be a minimum number of temperature recordings, ideally measured continuously. Right now, patients are sent home with a thermometer (and asked to record) every 4 hours while awake.

[Interviewee 6]

Monitoring strategies were noted to be variable and context dependent. For example, onset of CRS in CAR T-cell recipients typically occurs 4‐6 days after infusion, compared to earlier onset with bispecific therapies. Concerns were raised about the reliability of outpatient self-measurements and infrastructure to reliably implement outpatient monitoring at scale.

#### Theme 3: Balancing CRS Outpatient Monitoring With Patient and Caregiver Experience

Outpatient therapy requires the ability to effectively monitor key measures for CRS, including temperature, heart rate, blood oxygen saturation, and blood pressure. Patients often lack the energy to track symptoms and adhere to monitoring schedules, and caregivers frequently assume responsibility for tasks like symptom recording. Technology is seen as highly valuable in this context by helping to capture and share data with caregivers and care teams.

Given during the night, this is where DHTs shine… The patient and caregivers can rest with ease.

[Interview 1]

Clear messaging is critical, as patients need guidance on when to return to the hospital without undue alarm; the narrow window for triage and treatment is particularly important for patients who live far from care centers.

#### Theme 4: Reliable Early Warning Requires Context-Rich, Interoperable Data

Effective early warning for CRS relies on robust, interoperable data systems that capture not just values but also their context. Accurate interpretation of data depends on metadata that capture variables like sensor location, patient positioning, medication administration, and therapy timing.

One of the things that stood out was getting to the nuances of how we can line up the data. It’s important to capture when variables change, the timing relative to therapy, and the sequence of events.

[Interviewee 4]

Data standards like Fast Healthcare Interoperability Resources are strongly recommended to ensure interoperability and facilitate integration across technologies and settings. Implementing these systems for CRS in the outpatient setting requires infrastructure that supports consistent, high-quality data collection, including guidance on measurement methods, intervals, and operational procedures.

Collectively, findings from the literature review, survey, focus group, and expert interviews indicate the measures most informative as early warning signs of CRS. Across the 4 themes, patterns emerged regarding the clinical relevance, reliability, and practical implementation of the physiological signs, laboratory markers, and clinical symptoms. These findings informed the development of a consensus-derived common set of early warning signs for CRS.

A common set of early warning signs for CRS was established by identifying the minimum data required for the development of an early warning model for CRS. Four key measures—fever, hypoxia, tachycardia, and hypotension—were recognized as essential through the survey, whereas all remaining measures were selected in fewer than 25% of responses. The SMEs also supported the feasibility of digitally capturing these measures, with over 80% of responses indicating these measures can be derived digitally using currently available technology. KOLs similarly selected temperature, SpO_2_, heart rate, and blood pressure as the measures with the greatest likelihood of informing the development of an early warning model for CRS. The goal of the common set of early warning signs for CRS is to facilitate the collection of standard measures, which includes a minimum measurement frequency. Such a standardized approach, which is the focus of the ontology, is necessary to assemble fit-for-purpose datasets. In addition, it enables the evaluation of the early warning value of measure features for detecting CRS by defining measurement concepts, properties, and values.

### An Ontology to Guide the Collection of Fit-for-Purpose Datasets and Model Development

The synthesis of the results was performed with the goal of identifying the parameters most strongly related to impending CRS during the early, posttreatment phase. An ontology was developed for CRS by incorporating the selected measures to begin the effort toward standardization in data collection. Impending CRS is characterized by an early warning sign, defined as a change in a measure over a period of time before a critical transition to a clinically diagnosable disease state, which is classified into primary or secondary measures, each with associated metadata.

**Figure 4.**
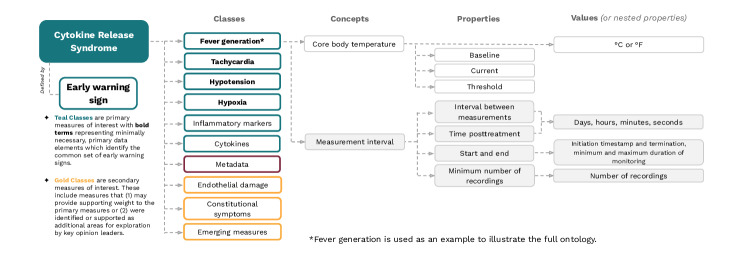
Ontology of early warning signs for CRS. This figure presents an ontology organizing early warning signs associated with CRS. CRS: Cytokine Release Syndrome.

Metadata included in the ontology is critical for characterizing the CRS response in relation to patient-specific features and managing the collected data. This information includes individual-specific variables (eg, age), environmental variables (eg, location of use), context-of-use variables (eg, disease), and measurement modality (eg, form factor). Classes of primary and secondary measures include information on specific concepts, properties, and their values. In general, concepts include selected measures and their measurement interval. Instead of introducing all concepts with their properties and values, the ontological structure is demonstrated using fever generation (Figure 4, Tables3  4).

## Discussion

### Principal Findings

This study presents an ontology of early warning signs for CRS designed to promote the uniform collection of high-frequency data, aiding the development of early warning models for CRS. To support this effort, a common set of digitally-derivable early warning signs for CRS (fever generation, tachycardia, hypotension, and hypoxia) was identified that facilitates data aggregation and promotes the evaluation of their early warning value for CRS.

The ontology provides measures that can be readily assessed with widely available technologies in inpatient, outpatient, and hospital-at-home settings (eg, temperature, heart rate, and other vital signs) as well as measures requiring specialized tests that may not be broadly available (eg, blood concentration of interleukin cytokines) (Tables3  4) [12]. The ontology, therefore, provides a framework for applying DHTs in practice, enabling continuous, real-time data collection that complements established CRS grading systems (eg, ASTCT criteria), ensuring alignment with current clinical workflows while expanding to outpatient settings. It includes both objective (eg, DHT-based) and subjective (eg, self-reported) measures. The latter offers corroboration of objective measures, often including high-frequency data collection, and provides a way to include the patient’s lived experiences within their care journey [61].

A key strength of the ontology is its ability to incorporate future measures as scientific understanding and technology evolve, ensuring ongoing relevance and usage. This design accommodates the future development of validated sensor-based DHTs to capture existing measures and for supporting validation of novel digital measures that have yet to be developed. The latter may provide objective data for subjective measures such as fatigue and chills (Table 4).

### Common Set of Early Warning Signs for CRS

The identified common set of early warning signs for CRS—fever generation, hypotension, hypoxia, and tachycardia— represents a minimum set of measures to facilitate the development of models for early warning of CRS. Fever is a hallmark feature of CRS and defines the diagnosis of CRS once possible other causes of fever have been ruled out [62]. Likewise, hypoxia and hypotension are key components of CRS grading scales used to assess severity based on low oxygen levels and low blood pressure, respectively [12]. Tachycardia during CRS is described as a rise in heart rate above a personal normal baseline and acceptable threshold, typically defined as >100 beats per minute by most institutions, when there is no additional cause attributable to the increase, such as physical activity and emotional stress [63]. While heart rate is not part of common CRS grading scales, heart rate values and trends may reinforce other measures within the common set. Our approach focuses on the most impactful indicators, which can be captured remotely to support outpatient care, rather than forcing the use of an exhaustive set of measures.

### Standardizing Data Collection for the Early Warning Signs for CRS Is Key to Advancing Data-Driven Tools for CRS

To support reproducible and interoperable data collection for CRS early warning, we outline clear guidance on measurement intervals, clinically meaningful thresholds, and quality control procedures below.

The process for measures collection should be well-defined with clear instructions, established frequency, timing and conditions, and specified quality controls and data handling procedures. The early warning signs for CRS are structured with properties and values to ensure unified reporting of data elements. We suggest that the properties consist of (1) the measure with an estimate of a patient’s baseline value, which may be outside of the normal range, current value, and clinically meaningful threshold value, and (2) the interval of measure, including the data capture period and interval between readings.

The data capture period should extend at least 2 SDs from the median onset time of CRS and continue until the patient is no longer within the data capture period or, at minimum, for 24 hours after the onset of CRS symptoms [64]. Likewise, the common set of early warning signs should be collected at least every 4 hours during the patient’s waking hours, with additional data collected during an event trigger (eg, feeling ill) clearly labeled to indicate it falls outside the standard collection frequency.

Accurate documentation of metadata is critical for contextualizing the early warning signs of CRS. This review has highlighted that variables such as immunotherapy type, disease burden, number and type of prior and concomitant treatments, and the administration of supportive care (ie, drugs like acetaminophen, tocilizumab, and dexamethasone; oxygen supplementation) serve as essential metadata and provide critical context for interpreting the common set of measures. Individual-level characteristics such as age, sex, and disease burden are important for understanding the broader clinical implications and their relationship to CRS onset and severity. Context-of-use data such as the cancer type and interventions (eg, steroids) can aid in understanding how differences in treatment strategies and management of adverse events impact CRS. Environmental variables may play vital roles in defining subpopulations to enhance model predictions.

Reporting data on measurement modalities supports quality control procedures, transparency in data collection methods, and comparison of sensor technology for potential influences on collected data. The accurate documentation of these metadata is at least as important as the common set of measures themselves, as they will often form the reference standard against which algorithms are trained and validated. To ensure these models are robust and generalizable, training must be performed on large, diverse datasets that reflect real-world patient variability across demographics, disease states, and clinical settings.

The properties of each measure (eg, baseline, threshold, collection intervals) offer varying levels of value. Baseline data provides a reference for understanding individual-level changes over time, offering the ability to improve early warning models. Collecting at least 24 hours of baseline data ensures a direct comparison over time, accounting for natural daily fluctuations, such as those driven by circadian rhythms [65]. Since CRS typically occurs more than 24 hours after immunotherapy, high-frequency collection of these measures may begin on the day of therapy administration. However, additional baseline monitoring prior to treatment is encouraged, as it may provide valuable insights into early signs and trends leading up to CRS onset.

Furthermore, thresholds for the measures may vary across institutions and between patients. While a set threshold value for fever is commonly agreed upon (38°C or 100.4°F) for grading CRS, there is currently no defined threshold for the other early warning signs of CRS [12]. It is critical to report the thresholds to ensure accurate comparison between patients and sites for CRS grading and management and support the collection of a representative population that may otherwise be constrained by exclusion criteria that will support building more personalized representative models based on a diverse population [12]. Physiologic baselines can differ significantly between individuals due to factors such as age, disease status, medication use, and comorbidities. As such, population-level thresholds may not adequately capture meaningful clinical changes for all patients. Establishing personalized baselines is critical to detect deviations that are clinically significant for a given individual and will improve both the accuracy and sensitivity of early warning models.

Finally, the current standard of care sets the minimum requirements for data collection. For example, following the gold standard of inpatient practice, the common set of measures for CRS should be collected at least every 4 hours, with a minimum of 4 readings per day. However, to develop high-quality models, more frequent data is likely necessary. Passive and continuous collection of these measures using fit-for-purpose DHTs offers an opportunity to meet this need.

The consistency across measures supports organized and structured data collection, which may simplify workflows, particularly for developing multimodal algorithms that will likely hold the highest sensitivity and specificity for the complex and variable nature of CRS [66-68]. While the current grading systems are based on multimodal but sporadic data, continuous monitoring of these measures, combined with data analytics and statistics, will provide data-driven insights that may help identify truly predictive features by grounding them in real-time, continuous data rather than static observations. Binning continuously collected data into one-hour intervals and reporting summary statistics (eg, minimum, maximum, median, and IQR) will enhance data availability, facilitate easier sharing and integration, and support data quality control.

Where available, the common set of early warning signs for CRS and their values also align with common content, terminology, and transport standards [69-71]. Structuring data according to Fast Healthcare Interoperability Resources, for example, supports integration of continuous monitoring data from sensor-based DHTs with clinical lab and electronic health record data, creating harmonized datasets suitable for training predictive models. Initial modeling efforts might focus on simpler, interpretable approaches such as decision trees and logistic regression to establish baseline performance, with opportunities to explore more sophisticated methods, including ensemble learning and time-series models, to capture complex temporal patterns in physiological data. This alignment facilitates data integration across platforms and will support the future development of more advanced early warning models for CRS leveraging machine learning and artificial intelligence technologies.

Importantly, these approaches also serve as the foundation for formal evaluation of the predictive value of CRS early warning signs. Future studies could systematically validate these measures by comparing model-based predictions against observed CRS onset and severity across diverse patient cohorts, assessing performance metrics as appropriate. Established methodologies such as the verification, analytical validation, and clinical validation (V3) framework, which offers a structured approach for validating sensor-based DHTs, and the Predictive Biomarker Modeling Framework which applies contrastive learning to systematically identify and assess predictive biomarkers, may guide this process [72,73]. Leveraging such frameworks would enable systematic assessment of predictive value and support the development of clinically relevant early warning models. Achieving reliable model performance will also depend on access to diverse datasets, which are essential for training algorithms that can generalize across patient populations and care environments.

### The Ontology of Early Warning Signs for CRS Provides Space for Technological and Scientific Advancements for Future Inclusion of New and Novel Digital Measures

At present, the development and severity of CRS following cancer-targeted immunotherapies is assessed using clinical judgment. Clinical trial results for each therapeutic agent, including dose and dosing schedule, offer guidance on the mean time to onset, duration, and percentage of incidence and severity for CRS [74]. Furthermore, tumor burden and overall health status also inform clinical decision processes [38]. Despite these valuable observations, there are currently no validated predictive models for CRS incidence, onset, or severity.

The ontology for early warning signs for CRS provides a foundation to establish data-driven models for CRS. Monitoring the common set of early warning signs for CRS, which are all physiological indicators, can be monitored remotely [75]. Continuous monitoring of temperature, SpO_2_, and heart rate with DHTs greatly enhances the ability to track clinical status through objective physiologic changes. Indeed, tracking temperature using a wearable (eg, a patch or strap) temperature sensor may detect the onset of CRS several hours earlier than standard of care with the potential for additional improvements when using personalized data and thresholds [15,65]. In the future, using continuous measurements by DHTs to characterize a patient’s baseline measurements could permit the identification of CRS using rules based on changes from baseline and trends, in addition to a commonly accepted threshold (ie, 38°C).

Laboratory markers, including levels of interleukins, CRP, ferritin, platelets, and lactate dehydrogenase, on the other hand, endure time delay in processing and testing availability, particularly for cytokine measurements that may be more well defined and more foundationally central to identifying CRS but are not commonly measured in the community hospital setting or in the outpatient setting. These data reflect a fixed time point that may miss dynamic transitions or rates of change critical for identifying early warning signs of CRS [11,46]. These barriers to care may be resolved over time through scientific and technological advancements such as the development of continuous monitoring technologies of these laboratory markers or advancements toward affordable and widespread point-of-care testing. For example, devices are being developed to continuously monitor cytokine levels in real-time from interstitial fluid and sweat; however, improvements are needed to enhance specificity for individual cytokines [76,77].

There are also challenges associated with studying the symptomatology in the form of patient-reported outcomes (PROs) for CRS. Presenting clinical symptoms may be directly due to emerging CRS or attributed to underlying disease conditions, other treatments with their own adverse event profiles, or overlap with various comorbidities. Furthermore, symptoms can be difficult to identify or lack congruence between patients due to the subjective nature of the measure (eg, reporting malaise when feeling extremely tired instead of fatigue, variable presentation of symptoms based on treatment or personal factors). Regular self-reporting of symptoms via an app or another DHT could be valuable to improve consistency, and the occurrence or severity of specific symptoms such as chills could be integrated with the other outcome measures to inform predictive models.

Here, we have identified a preliminary set of symptoms that commonly occur before and up to the onset of CRS. Incorporating measures of symptoms into the data model permits the identification of symptom or groups of symptoms with defining features that are capable of producing new evidence for the advancement of early warning signs for CRS [78]. With no established PROs or observer-reported outcomes specific to CRS, it is important to critically assess and intentionally implement targeted assessments to minimize the burden to patients, caregivers, and clinical staff [79]. For instance, modification of the PRO-CTCAE for cancer clinical trials to allow for real-time reporting using the symptoms defined within the early warning signs ontology for CRS offers a validated open-access platform for creation of cancer-related PROs, which can be digitally deployed to support outpatient care and decentralized clinical trials [80]. Limitations of PROs (eg, recall bias, compliance, and data completion), however, reduce the probability of independently using PROs as an early warning sign for CRS [81]. As such, there is a growing need for novel digital clinical measures that can be continuously captured in real-time by sensor-based DHTs to objectively and passively define the symptomatology associated with CRS.

Our ontology provides an overview of the current state of early warning signs for CRS. Moving forward, more clinical trials will be needed to train and validate predictive algorithms across diverse cohorts, followed by randomized controlled trials to assess their feasibility and effectiveness in outpatient settings. Ideally, the DHTs will be noninvasive; for example, monitoring could involve wearable form factors, such as a chest strap or wristband, allowing patients to recover at home rather than in a hospital. This will contribute to ensuring patients’ compliance with the selected technology. Importantly, future implementation will require regulatory validation and seamless integration into existing clinical workflows. Additionally, a similar approach could be applied to other adverse events, such as ICANS. Given the significant clinical overlap between CRS and ICANS, which includes shared symptoms and treatment strategies, it is important to recognize that ICANS frequently follows CRS [12]. Developing a comprehensive ontology that integrates early warning signs for both conditions could improve predictive accuracy and facilitate more coordinated clinical decision-making. When combined with an early warning model for CRS, this could contribute to a comprehensive remote monitoring system for patients undergoing immunotherapy, better reflecting real-world management where these conditions often coexist.

### Limitations

This scoping review did not consider predictive values outside of the defined time window, which may limit the generalizability of the findings to broader prediction-based models (eg, patient stratification prior to immunotherapy, patient stratification after development of CRS). Additionally, gray literature was not included in the review, which could contain valuable insights, particularly in this early stage of the field where much relevant information has yet to be published in peer-reviewed publications. The analysis was deductive, based on literature-derived measures, which provided a solid foundation but could limit the emergence of novel concepts. This informed the decision to include KOL interviews to supplement the review. Future research should consider expanding evidence sources to include gray literature such as preprints, ongoing clinical trials, and unpublished datasets. This requires systematic search strategies, quality appraisal, and transparent reporting to ensure comprehensive and reliable synthesis while minimizing duplication and bias.

Another limitation is the potential variability in CRS, adjudication, diagnostic criteria, and grading scales across studies, which may affect the consistency and comparability of findings, highlighting the need for standardized definitions in future digital health research. Furthermore, the review did not evaluate the actual predictive value of the measures, which remains a critical area for future research as outlined in the discussion. As the field moves toward more data-driven and decentralized approaches to CRS management, future systematic reviews and meta-analyses should aim to integrate data from both traditional and real-world sources, stratify findings by CRS grading frameworks, and assess heterogeneity in measurement modalities and data collection contexts. Such approaches would enhance the interpretability, reproducibility, and clinical relevance of predictive model development.

### Future Directions

Identifying early warning biomarkers of CRS will rely on high-quality, fit-for-purpose data from the intended-use population and sufficiently large, diverse datasets to develop and validate algorithms. Several laboratory-based markers are being evaluated for predicting the occurrence, onset, and severity of CRS [52,82], but these markers are currently restrictive, especially for postimmunotherapy early warning, due to the processing time, limited number of data points available, and blood draws that normally require the patient to remain inpatient or otherwise involved in an intensive posttreatment monitoring program with frequent clinic visits.

Establishing predictive biomarkers, especially those that can be continuously and passively collected, represents a key future direction, as these biomarkers may mitigate the risks associated with CRS following cancer-targeted immunotherapies. Realizing this potential will require strong collaboration among clinicians, researchers, and technology developers to ensure comprehensive data collection, model development, and clinical integration. We encourage the collection of the early warning signs identified in the ontology and the adoption of the recommended primary and secondary measures of interest.

### Conclusions

This ontology of early warning signs for CRS establishes a guide for building fit-for-purpose datasets from measures collected prior to the onset of CRS. These datasets will play an important role in advancing the development of early warning models and ultimately support decentralized clinical trials and outpatient treatment options. Consistent data collection will facilitate the training and validation of early warning models for CRS while remaining easy to adopt and integrate into clinical research and practice. Understanding the early factors that occur before the onset of CRS is paramount for developing diagnostic and predictive models for CRS onset and severity, and these data elements may contribute to more equitable and consistent access to these immunotherapies.

## Supplementary material

10.2196/71956 Multimedia Appendix 1 Supplemental methods: search strategy, survey, and interview guides.

10.2196/71956 Multimedia Appendix 2 Characteristics of the studies eligible for inclusion in the scoping review.

10.2196/71956 Checklist 1 PRISMA-ScR checklist.

## Abbreviations

ASTCT: American Society for Transplantation and Cellular Therapy
CAR T-cell: Chimeric Antigen Receptor T-cell
CARTOX: CAR T-Cell Therapy–Associated TOXicity
CRP: C-reactive protein
CRS: cytokine release syndrome
CTCAE: Common Terminology Criteria for Adverse Events
DATAcc: Digital Health Measurement Collaborative Community
DHT: digital health technology
DiMe: Digital Medicine Society
ICANS: immune effector cell-associated neurotoxicity syndrome
KOL: key opinion leader
PICO: population, intervention, comparator, and outcome
PRISMA: Preferred Reporting Items for Systematic Reviews and Meta-Analyses
PRO: patient-reported outcome
PROSPERO: International Prospective Register of Systematic Reviews
SME: subject matter expert
SpO2: saturation of peripheral oxygen
