# Supplementary material for: Digital Biomarkers of Cytokine Release Syndrome: Scoping Review and Ontology Development of the Role and Relevance of Digital Measures Using a Mixed Methods Approach
Source: J Med Internet Res. 2025 Dec 11;27:e71956. doi: 10.2196/71956 (PMC12699293; doi:10.2196/71956)
Supplement: Multimedia Appendix 1 [file jmir-v27-e71956-s001.docx]

**Search strings**

**Pubmed**

Complete search string returning 221 results as of March 19st, 2024:

((((Humans[MeSH Terms]) AND (Cytokine release syndrome OR cytokine storm)) AND (immunotherapy OR Immunotherapy, Adoptive[MeSH Terms])) AND (Sign*[tiab] OR Symptom*[tiab] OR biomarker*[tiab] OR measure*[tiab])) AND (Onset*[tiab] OR Early*[tiab] OR predict*[tiab] OR incidence*[tiab]) AND (2014/01/01:2024/03/30[pdat])

Search tags in square brackets are as follows: mesh = Medical subject headings; tiab = Title and abstract*; pdat = Publication date.

* Keywords are searched within the citation title, collection title, abstract, and author keywords.

**Embase**

Complete search string returning 57 results as of March 19st, 2024:

('cytokine release syndrome' OR 'cytokine storm') AND ('immunotherapy' OR 'adoptive immunotherapy') AND (human) AND (sign*:ti,ab OR symptom*:ti,ab OR biomarker*:ti,ab OR measure*:ti,ab) AND (onset*:ti,ab OR early*:ti,ab OR predict*:ti,ab)

**Survey**

**/// SURVEY PAGE 1 ///**

Please provide your name, organization, and area of expertise, below. Kindly note that only anonymized findings will be presented to the group.

- *[Open text]*

**/// SURVEY PAGE 2 ///**

**Signs**

Please indicate whether or not you would recommend the following measures to be included in a common set of measures that may be used to develop a risk prediction model for CRS. What other measures or considerations need to be taken into account?

| **Proposed Digital Measure** | **Units** | **Type of Measure** |
| --- | --- | --- |
| Temperature | °C or °F | Physiological |
| Oxygen Saturation | % (SpO2) | Physiological |
| Respiratory Rate | # of breaths per minute | Physiological |
| Heart Rate | Beats per min | Physiological |
| Blood Pressure | mm Hg | Physiological |
| *Heart Rate Variability* | *milliseconds (ms), defined interval* | *Physiological* |

*[1.1]* *multiple choice*

**Measure:** Core body temperature (fever generation)

1. Yes, I would recommend.
2. No, I would not recommend.
3. I need more information.

*[1.2]* *open text*
Please provide your reasoning or any notes you would like to add about measuring **core body temperature (fever generation)**.

*[2.1]* *multiple choice*

**Measure:** Oxygen Saturation

1. Yes, I would recommend.
2. No, I would not recommend.
3. I need more information.

*[2.2]* *open text*
Please provide your reasoning or any notes you would like to add about measuring **oxygen saturation**.

*[3.1]* *multiple choice*

**Measure:** Respiratory rate

1. Yes, I would recommend.
2. No, I would not recommend.
3. I need more information.

*[3.2]* *open text*
Please provide your reasoning or any notes you would like to add about measuring **respiratory rate**.

*[4.1]* *multiple choice*

**Measure:** heart rate

1. Yes, I would recommend.
2. No, I would not recommend.
3. I need more information.

*[4.2]* *open text*
Please provide your reasoning or any notes you would like to add about measuring **heart rate**.

*[5.1]* *multiple choice*

**Measure:** blood pressure

1. Yes, I would recommend.
2. No, I would not recommend.
3. I need more information.

*[5.2]* *open text*
Please provide your reasoning or any notes you would like to add about measuring **blood pressure**.

*[6.1]* *multiple choice*

**Measure:** heart rate variability *note: This was recommended by SME, not found in peer-reviewed literature

1. Yes, I would recommend.
2. No, I would not recommend.
3. I need more information.

*[6.2]* *open text*
Please provide your reasoning or any notes you would like to add about measuring **heart rate variability**.

**/// SURVEY PAGE 3 ///**

**Laboratory biomarkers**

Please indicate whether or not you would recommend the following measures to be included in a common set of measures that may be used to develop a risk prediction model for CRS. What other measures or considerations need to be taken into account?

| **Proposed Digital Measure** | **Units** | **Type of Measure** |
| --- | --- | --- |
| Cytokines | Mass/volume (pg/mL) | biochemical |
| C-reactive Protein | Mass/volume (mg/dL) | biochemical |
| Ferritin | Mass/volume (ng/mL) | biochemical |
| Coagulation factors (coagulation factor test, albumin, plasminogen activator inhibitor-1) | Seconds or INR, Mass/volume (g/dL), Mass/volume (ng/mL) | biochemical |
| EASIX score (lactate dehydrogenase, platelet) | Units/Liter; counts/Liter | biochemical |

*[1.1]* *multiple choice*

**Measure:** Cytokines

1. Yes, I would recommend.
2. No, I would not recommend.
3. I need more information.

*[1.2]* *open text*
Please provide your reasoning or any notes you would like to add about measuring **cytokines**.

*[2.1]* *multiple choice*

**Measure:** c-reactive protein

1. Yes, I would recommend.
2. No, I would not recommend.
3. I need more information.

*[2.2]* *open text*
Please provide your reasoning or any notes you would like to add about measuring **c-reactive protein**.

*[3.1]* *multiple choice*

**Measure:** Ferritin

1. Yes, I would recommend.
2. No, I would not recommend.
3. I need more information.

*[3.2]* *open text*
Please provide your reasoning or any notes you would like to add about measuring **ferritin**.

*[4.1]* *multiple choice*

**Measure:** coagulation factors

1. Yes, I would recommend.
2. No, I would not recommend.
3. I need more information.

*[4.2]* *open text*
Please provide your reasoning or any notes you would like to add about measuring **coagulation factors**.

*[5.1]* *multiple choice*

**Measure:** EASIX-related measures

1. Yes, I would recommend.
2. No, I would not recommend.
3. I need more information.

*[5.2]* *open text*
Please provide your reasoning or any notes you would like to add about measuring **EASIX-related measures**.

**/// SURVEY PAGE 4 ///**

**Symptoms**

Please indicate whether or not you would recommend the following measures to be included in a common set of measures that may be used to develop a risk prediction model for CRS. What other measures or considerations need to be taken into account?

| **Proposed Digital Measure** | **Units** | **Type of Measure** |
| --- | --- | --- |
| Malaise | Timestamp, scale | symptom |
| Chills | Timestamp | symptom |
| Fatigue | Timestamp, scale | symptom |
| rash | Timestamp, description | symptom |
| confusion | Timestamp, score | symptom |
| Dyspnea (shortness of breath) | Timestamp, scale | symptom |
| Reduced appetite | Timestamp, scale | symptom |
| headache | Timestamp, scale | symptom |
| nausea/vomiting | Timestamp, scale | symptom |
| myalgia | Timestamp, scale | symptom |

*[1.1]* *multiple choice*

**Measure:** Malaise

1. Yes, I would recommend.
2. No, I would not recommend.
3. I need more information.

*[1.2]* *open text*
Please provide your reasoning or any notes you would like to add about measuring **malaise**.

*[2.1]* *multiple choice*

**Measure:** chills

1. Yes, I would recommend.
2. No, I would not recommend.
3. I need more information.

*[2.2]* *open text*
Please provide your reasoning or any notes you would like to add about measuring **chills**.

*[3.1]* *multiple choice*

**Measure:** Fatigue

1. Yes, I would recommend.
2. No, I would not recommend.
3. I need more information.

*[3.2]* *open text*
Please provide your reasoning or any notes you would like to add about measuring **fatigue**.

*[4.1]* *multiple choice*

**Measure:** rash

1. Yes, I would recommend.
2. No, I would not recommend.
3. I need more information.

*[4.2]* *open text*
Please provide your reasoning or any notes you would like to add about measuring **rash**.

*[5.1]* *multiple choice*

**Measure:** confusion

1. Yes, I would recommend.
2. No, I would not recommend.
3. I need more information.

*[5.2]* *open text*
Please provide your reasoning or any notes you would like to add about measuring **confusion**.

*[6.1]* *multiple choice*

**Measure:** dyspnea (shortness of breath)

1. Yes, I would recommend.
2. No, I would not recommend.
3. I need more information.

*[6.2]* *open text*
Please provide your reasoning or any notes you would like to add about measuring **dyspnea (shortness of breath)**.

*[7.1]* *multiple choice*

**Measure:** reduced appetite

1. Yes, I would recommend.
2. No, I would not recommend.
3. I need more information.

*[7.2]* *open text*
Please provide your reasoning or any notes you would like to add about measuring **reduced appetite**.

*[8.1]* *multiple choice*

**Measure:** headache

1. Yes, I would recommend.
2. No, I would not recommend.
3. I need more information.

*[8.2]* *open text*
Please provide your reasoning or any notes you would like to add about measuring **headache**.

*[9.1]* *multiple choice*

**Measure:** nausea/vomiting

1. Yes, I would recommend.
2. No, I would not recommend.
3. I need more information.

*[9.2]* *open text*
Please provide your reasoning or any notes you would like to add about measuring **nausea/vomiting**.

*[10.1]* *multiple choice*

**Measure:** myalgia (muscle pain)

1. Yes, I would recommend.
2. No, I would not recommend.
3. I need more information.

*[10.2]* *open text*
Please provide your reasoning or any notes you would like to add about measuring **myalgia (muscle pain)**.

**/// SURVEY PAGE 4 ///**

*[]* *open text*
Are there additional measures that, when collected after immunotherapy and up to CRS diagnosis, may be predictive of the onset or progression of CRS that you would like to recommend? Please list them below.

*[]* *select all that*

Of the previously identified signs, symptoms, and biomarkers, which measures related to early warning/monitoring for onset of CRS do you agree can be digitally-derived with currently available technology in an outpatient setting?

1. Temperature (Fever generation)
2. Heart rate
3. Blood pressure
4. Oxygen saturation (hypoxia)
5. Respiratory rate
6. Cytokines
7. Inflammatory marker [C-reactive protein]
8. Inflammatory marker [ferritin]
9. Coagulation/ fibrinolysis parameters (albumin, Plasminogen activator inhibitor 1 (PAI-1), fibrinogen)
10. Malaise
11. Chills
12. Fatigue
13. Rash
14. Confusion
15. Dyspnea
16. Reduced appetite
17. Headache
18. nausea/vomiting
19. Myalgia
20. None of the above
21. Other *[]* *open text*

*[]* *select all that*

Of the previously identified signs, symptoms, and biomarkers, which early warning/monitoring for onset measures, if any, do you agree are minimally necessary or required for inclusion into a risk prediction model of CRS for use in an outpatient setting?

1. Temperature (Fever generation)
2. Heart rate
3. Blood pressure
4. Oxygen saturation (hypoxia)
5. Respiratory rate
6. Cytokines
7. Inflammatory marker [C-reactive protein]
8. Inflammatory marker [ferritin]
9. Coagulation/ fibrinolysis parameters (albumin, Plasminogen activator inhibitor 1 (PAI-1), fibrinogen)
10. Malaise
11. Chills
12. Fatigue
13. Rash
14. Confusion
15. Dyspnea
16. Reduced appetite
17. Headache
18. nausea/vomiting
19. Myalgia
20. None of the above/not enough information to establish any measure as a required for inclusion into a risk prediction model for CRS
21. Other *[]* *open text*

*[]* *open text*
Are there any general comments you’d like to make or anything we should clarify?

End of Survey

**Interview topic guide**

- Introductions & brief project overview
  - Provide project goals and purpose of the interview
  - Describe our use of the recording and how interview findings will inform deliverables
  - Describe project deliverables (open-access toolkit & manuscript)
  - Outline project outputs and next steps (analysis, workshop & manuscript)
  - Obtain informed consent
- Briefly describe and review pre-read materials
  - Review via screenshare the PDFs
  - Gather their initial reactions
- Conduct a gap analysis and identify what may be incorrectly portrayed on select resources
  - *Focus #1*: Measures of CRS and their values
  - *Focus #2*: Definitions of key terms
- Measures of CRS and their values
  - Vital Signs
  - Symptoms
  - Laboratory markers
- Key terminology
  - Evaluate definitions for:
    - CRS
    - Early Warning Signals
    - Severe CRS
  - Explore current Standard of Care for CRS
- Conclusion
  - Thank participants for their time and feedback
