# Supplementary material for: Digital Biomarkers of Cytokine Release Syndrome: Scoping Review and Ontology Development of the Role and Relevance of Digital Measures Using a Mixed Methods Approach
Source: J Med Internet Res. 2025 Dec 11;27:e71956. doi: 10.2196/71956 (PMC12699293; doi:10.2196/71956)
Supplement: Multimedia Appendix 2 [file jmir-v27-e71956-s002.docx]

Table S1. Characteristics of the studies eligible for inclusion in the scoping review

| Author & date | Disease | Treatment type | Measure(s) reported | CRS time to onset, day mean (range) | Sample size | Age, years range | Grading scale(s) | severeCRS grade |
| --- | --- | --- | --- | --- | --- | --- | --- | --- |
| Abboud et al. 2016 [[30]](https://paperpile.com/c/2YIamu/BhOs) | acute myeloid leukemia (AML); acute lymphoblastic leukemia (ALL); myelodysplastic syndromes (MDS); Lymphoma; Aplastic anemia; leukemia (other) | G-CSF mobilized T-cell replete peripheral blood haploidentical hematopoietic cell transplantation (haplo-HCT) | temperature; oxygen requirement (FiO2); cytokines [G-CSF, GM-CSF, IFNα2, IFNγ, IL-10, IL-12P70, IL-13, IL-15, IL-17A, IL-1β, IL-2, IL-3, IL-4, IL-5, IL-6, IL-8, MCP-1, TNF-α] | - | 75 | 19-73 | Lee | 3 |
| Caimi et al. 2021 [[31]](https://paperpile.com/c/2YIamu/YpFH) | relapsed non–Hodgkin lymphoma (NHL) including Diffuse large B cell lymphoma (DLBCL), Transformed follicular lymphoma, Mantle cell lymphoma, and Burkitt lymphoma | tisagenlecleucel or investigational antiCD19 CAR T-cells | cytokines [TNF-ɑ, TGF-β3, TGF-β2, TGF-β1, SDF-1ɑ, MIP-3β, MIP-3ɑ, MIP1β, MIP-1ɑ, MCP-3, MPC-1; ITAC, INF-γ, IL-33, IL-29, IL-27, IL-22, IL-21, IL-18, IL-17ɑ, IL-15, IL-10, IL-9, IL-8, IL-7, IL-6, IL-4, IL-1β, Fractalkine] | (3-7) | 20 | 33 – 76 | Lee | 3 |
| Davis et al. 2023 [[32]](https://paperpile.com/c/2YIamu/ZUo7) | non-Hodgkin lymphoma (NHL) including DLBCL, MCL, and FL; acute lymphoblastic leukemia (ALL); multiple myeloma (MM) | CAR T-cell therapy | temperature; lactate dehydrogenase (LDH); C-reactive protein (CRP); ferritin; IL-6 | (0-3) | 40 | 23-77 | ASTCT | 3 |
| Dimitriou et al. 2019 [[33]](https://paperpile.com/c/2YIamu/0gio) | BRAF V600 mutant melanoma | immune-checkpoint inhibitors and BRAF/MEKinhibitors (BRAFi/MEKi) | cytokine [IL-6, IFN-γ, and TNF-α]; CRP; rash; pyrexia; chills; tachycardia; hypotension | - | 2 | 47-48 | - | - |
| Diorio et al. 2022 [[34]](https://paperpile.com/c/2YIamu/euSX) | B-cell acute lymphoblastic leukemia (B-ALL) | CD19 directed CAR T-cell therapy, CTL019 | 1463 proteins [Olink Explore 1536/384 panel]; sC5b9 | - | 26 | 8-11.2 (*median age range*) | Penn | 3 |
| Dong et al. 2023 [[35]](https://paperpile.com/c/2YIamu/enhA) | relapsed/refractory multiple myeloma (MM) | B-cell  maturation antigen  (BCMA) CAR T-cell therapy | ALP (alkaline phosphatase); CRP; ferritin; cytokines (IL-6, IFN-γ) | 5.5 (1-10) | 27 | 43-70 | ASTCT | - |
| dos Santos et al. 2022 [[36]](https://paperpile.com/c/2YIamu/LnVw) | R/R large B-cell lymphoma (LBCL); B-cell precursor acute lymphoblastic leukemia (BCP-ALL); mantle cell lymphoma (MCL) | axicabtagene ciloleucel (axi-cel); tisagenlecleucel (tisa-cel); KTE-X19 | CRP; IL-6; ferritin | (0-14) | 64 | 19-82 | ASTCT | 2 |
| Gardner et al. 2019 [[37]](https://paperpile.com/c/2YIamu/Cms5) | B-cell acute lymphoblastic leukemia (B-ALL), CD19+ leukemia | CD19 CAR T-cell product, SCRI-CAR19v1 | fever; CRP; ferritin; LDH; neutrophils; hemoglobin; lymphocytes; platelets; cytokines [granulocyte-macrophage colony-stimulating factor, TNF-α, INF-γ, IL-6, IL-2, IL-10, IL-5, IL-13, MIP-1b, sCD137, sFas, granzyme A, granzyme B] | (2-11) | 43 | 1-25 | CTCAEv4 | 4 |
| Hay et al. 2017 [[38]](https://paperpile.com/c/2YIamu/0Izk) | CD19+ relapsed/refractory B-cell acute lymphoblastic leukemia (B-ALL); chronic lymphocytic leukemia(CLL); non-Hodgkin lymphoma (NHL) | CD19 CAR T-cells | temperature; systolic blood pressure; diastolic blood pressure; heart rate; respiratory rate; weight; albumin; sera protein | (1.4-4.7) | 133 | 27-70 | Lee | 4 |
| Hoyt et al. 2024 [[39]](https://paperpile.com/c/2YIamu/o9pt) | B-cell acute lymphoblastic leukemia (B-ALL) | rapcabtagene autoleucel | Ferritin; CRP; Fever | 8 | 1 | 52 | ASTCT | - |
| Ishihara et al. 2022 [[40]](https://paperpile.com/c/2YIamu/DxC7) | NY-ESO-1-expressing solid tumors (breast, ovarian, Synovial sarcoma, Malignant salivary tumor, Melanoma, Myxoid liposarcoma) | TCR-transduced T-cell product (TBI-1301) | IL-6; monocyte chemotactic protein-1 (MCP-1)/CCL2; IL-3 | (1-2) | 9 | 40-70 | Lee | - |
| Kauer et al. 2020 [[41]](https://paperpile.com/c/2YIamu/rbpP) | castrate-resistant metastatic prostate carcinoma | bispecific PSMAxCD3 antibody CC-1 | IL-6; CRP; soluble IL-2 receptor (sIL-2R) levels; temperature | (4-6) | 3 | - | ASTCT | 3 |
| Knaus et al. 2022 [[42]](https://paperpile.com/c/2YIamu/hSG7) | Acute leukemia; myelodysplastic syndrome (MDS), myeloproliferative neoplasm (MPN), or overlap;  Lymphoma; Nonmalignant disease | Antithymocyte globulin (ATG)/anti-T lymphocyte globulin (ATLG) prophylaxis in hematopoietic stem cell transplantation (HSCT) | white blood cell count; platelets; prothrombin time ratio; CRP; creatinine; bilirubin; IL-6; procalcitonin; blood pressure; oxygen saturation; fever | - | 284 | 44-61 | ASTCT | 3 |
| Liu et al. 2023 [[43]](https://paperpile.com/c/2YIamu/2bVQ) | cell renal cell carcinoma, grade 2 | ipilimumab & nivolumab | temperature; tachycardia; chills; confusion; "feeling sick" | 0 | 1 | 70-75 | ASTCT | - |
| Moiseev et al. 2021 [[44]](https://paperpile.com/c/2YIamu/QPO5) | acute myelogenous leukemia (AML); acute lymphoblastic leukemia (ALL) | hematopoietic stem cell transplantation with post-transplantation bendamustine | ferritin; lactate dehydrogenase (LDH); cytokines [IL-6, IL-1β, IL-10, IL-17, INF-γ]; abnormal liver function [alanine aminotransferase (ALT), Aspartate Aminotransferase (​​AST), total bilirubin]; fever | 9 (1-98) | 27 | 20-56 | CARTOX, ASTCT | 3 |
| Napolitano et al. 2022 [[](https://paperpile.com/c/2YIamu/VZUA)45] | relapsed/refractory B-lineage acute lymphoblastic leukemia | tisagenlecleucel | temperature; heart rate; systolic blood pressure; diastolic blood pressure; respiratory rates; complete blood count; CRP; biochemistry; coagulation | 0.8 (0-3) | 5 | 3-21 | ASTCT, Penn, Lee | - |
| Pabst et al. 2020 [[46]](https://paperpile.com/c/2YIamu/c0Lk) | aggressive B-cell malignancies [acute lymphoid leukemia, diffuse large b-cell lymphoma, secondary DLBCL, follicular lymphoma, large B-cell lymphoma] | tisagenlecleucel; CAR017 CAR-T investigational product | IL-6; CAR-T transgene copy numbers | 3 (2-4) | 11 | 25-74 | Penn | 4 |
| Peaytt et al. 2023 [[47]](https://paperpile.com/c/2YIamu/FOtw) | acute lymphoblastic leukemia (ALL), non-Hodgkin’s lymphoma (NHL), and multiple myeloma (MM) | Cellular Immunotherapies (CART, NK, TIL, TCR); commercial or investigational immune effector cell therapy | CRP; ferritin; albumin; LDH; time of first fever; vital signs to grade CRS [temperature, blood pressure, oxygen saturation] | (2-5) | 28 | (*59-65 median age range*) | ASTCT | 3 |
| Pennisi et al. 2021 [[48]](https://paperpile.com/c/2YIamu/vfsO) | B-acute lymphoblastic leukemia (B-ALL); diffuse large B-cell lymphoma (DLBCL) | 1928z CAR T cells; axicabtagene ciloleucel; tisagenlecleucel | LDH; creatinine; platelets; CRP; fever | (0-12) | 118 | 20-86 | ASTCT | 3 |
| Sheng et al. 2023 [[49]](https://paperpile.com/c/2YIamu/H5VX) | diffuse large B-cell lymphoma (DLBCL) with active Sjögren’s disease (SjD) | axicabtagene ciloleucel | fever, fatigue; reduced appetite; vomiting; headache; muscular pain; IL-4; FN-γ; IL-17; IL-6; TNF-α | 1 | 1 | 76 | ASTCT | - |
| Shi et al. 2020 [[50]](https://paperpile.com/c/2YIamu/atSR) | hepatocellular carcinoma | CAR-GPC3 T cells | Hs-CRP; IFN-γ; IL-10; IL-15; IL-6; regulated upon activation, normal T cell expressed, and secreted (RANTES); monocyte chemoattractant protein (MCP)-1; temperature | - | 13 | 34-68 | Lee | 3 |
| Song et al. 2021 [[51]](https://paperpile.com/c/2YIamu/VRRB) | Hematologic cancer; Diffuse Large B-Cell  Lymphoma (DLBCL); Acute Lymphoblastic  Leukemia (ALL) | CAR-T therapy - any | IL-6; TNF-α; IL-2; MCP-1; IL-1β; IL-8; IL-10; IL-12; IL-17A; IFN-γ; ferritin; CRP; fever; hypotension; hypoxia | (1-9) | 10 | 21-75 | ASTCT | 2 |
| Teachey et al. 2016 [[52]](https://paperpile.com/c/2YIamu/qBHM) | acute lymphoblastic leukemia (ALL) with B-ALL, T-All with CD19 expression, primary refractory B-ALL | CTL019 CD19-directed therapy | 43 different cytokines, chemokines including IFN-γ, IL-6, IL-8, sIL2Rα, sgp130, sIL-6R, MCP1, MIP-1α, and GM-CSF; soluble receptors; ferritins; LDH; CRP; ALT; BUN; creatinine; fibrinogen; prothrombin time; partial thromboplastin time; fever; flu-like symptoms | 1 (0 – 10) | 51 | 5-72 | other | 3 |
| Topp et al. 2021 [[53]](https://paperpile.com/c/2YIamu/Cq2J) | relapsed or refractory large B-cell lymphoma (R/R LBCL) | axicabtagene ciloleucel | IFN-γ; IL-2; IL-6; IL-8; IL-15; MCP1; GM-CSF; CRP; ferritin | 2 (1–8) | 41 | 19–77 | Lee | 3 |
| Turtle et al. 2016 [[54]](https://paperpile.com/c/2YIamu/RHVa) | B cell acute lymphoblastic leukemia | CD19 CAR-T cells | IL-6; CRP; IFN-γ; ferritin; TNF-α; fever, hypotension | (0.25-9) | 29 | 20-73 | CTCAEv4 | - |
| Yamasaki-Morita et al. 2022 [[55]](https://paperpile.com/c/2YIamu/YhyO) | refractory/relapsed diffuse large B-cell lymphoma (DLBCL) | tisagenlecleucel | total plasminogen activator inhibitor 1; presepsin; sIL2R; fibrinogen; soluble fibrin; CRP; fever | 3 | 25 | 20–69 | ASTCT | 2 |
| Yang et al. 2024 [[56]](https://paperpile.com/c/2YIamu/OUox) | relapsed/refractory multiple myeloma (MM) | ciltacabtagene autoleucel | cytokines [CCL2, CCL3, CCL4, CCL5, CCL11, CCL19, CCL20, CX3CL1, CXCL1, CXCL2, CXCL10, IL-1α, IL-1β, IL-1ra, IL-2, IL-3, IL-4, IL-5, IL-6, IL-7, IL-8, IL-10, IL-12 p70, IL-13, IL-15, IL-17A, IL-17E, IL-33, EGF, FGF basic, VEGF, PDGF-AA, PDGF-AB/BB, TGF-α, G-CSF, GM-CSF, sPD-L1, sCD40 Ligand, sFLT-3Ligand, IFN-α, IFN-β, IFN-γ, TNF-α, TRAIL, Granzyme B, sIL-2Rα, CXCL9, gp130, sIL-1R II, sIL-6Rα, sTNF RI, sTNF RII, RAGE, angiopoietin-1, angiopoietin-2, IL-18, CCL22, sBCMA, sVEGF R1, sVEGF R2, sVEGF R3]; genetic profile; liver damage [aspartate aminotransferase (AST), alanine aminotransferase (ALT), fibrogen, bilirubin]; coagulation dysfunction [platelet count, activated partial thromboplastine time, fibrogen]; fever; hypotension; hypoxemia | 6 (1-10) | 26 | 35-73 | other | 3 |
| Zhao et al. 2024 [[57]](https://paperpile.com/c/2YIamu/dW9x) | acute myeloid leukemia (R/R AML) | CLL1 CAR-T cell therapy | creatinine; LDH; PLT; CRP | 3 (1-5) | 32 | 10-73 | ASTCT | 3 |
| Zheng et al. 2023 [[58]](https://paperpile.com/c/2YIamu/vXIB) | diffuse large B-cell lymphoma (DLBCL) stage IV A | sequential infusion CD22 & CD19 CAR T | IL-6; ferritin; fever; chills; shivering; headache; blood oxygen desaturation; shock; weakness; severe thirst; heart rate decline | 5 and 36 *(double CRS)* | 1 | 33 | - | - |
| Zhou et al. 2023 [[59]](https://paperpile.com/c/2YIamu/IH1O) | hematological malignancies : multiple myeloma (MM), acute lymphoblastic leukemia (ALL), non-Hodgkin lymphoma (NHL) | B-cell maturation antigen  (BCMA) CAR-T cell therapy | CRP, platelets, hemoglobin, WBC, creatinine, 45 cytokines [IL-12; IL-1β; IL-33; CD40L, IL-3; IL-15; IL-7; MIP-1β; GM-CSF; IL-10; G-CSF; GZMB; IL-1ra; PDL1; MIP1α; IL-6; CXCL-10; IFN-γ; TNF-α; CCL-11; TRAIL, VEGF; CXCL1; IL-8; MIP3β; MIP3α; Flt3L; MCP1; CX3CL1; EGF; CCL5; PDGFAA; PDGFAB; CXCL2; basic FGF; IFNβ; IL-17E; IL-1α; IL-13; TGF-α; INF-α; IL-4; IL-5; IL-17A; IL-2] | - | 214 | 30-65 | Lee | 3 |
